# Myasthenia Gravis Coexisting With Primary Sjögren's Syndrome: Report of Three Cases and Literature Review

**DOI:** 10.3389/fneur.2020.00939

**Published:** 2020-09-02

**Authors:** Xia Li, Yi Zhao, Qiuju Liao, Yuwei Da

**Affiliations:** ^1^Department of Rheumatology & Allergy, Xuanwu Hospital, Capital Medical University, Beijing, China; ^2^Department of Neurology, Xuanwu Hospital, Capital Medical University, Beijing, China

**Keywords:** myasthenia gravis, primary Sjögren's syndrome, autoimmune diseases, coexistence, outcome

## Abstract

**Objective:** The coexistence of myasthenia gravis (MG) and primary Sjögren's syndrome (pSS) is rarely reported. This study aims to describe the clinical features, treatment and outcome of MG coexisting with pSS.

**Materials and Methods:** Herein we reported three cases with the two coexisting diseases, and also searched the PubMed, Medline databases, and China Wanfang databases for the relevant case reports written in English, Chinese, or Japanese with detailed data.

**Results:** We reviewed a total of 17 patients with both diseases. Fifteen patients were female. The median age at onset was 48 years (range 28–78 years). MG was the initial disease in nine of 17 cases. The median interval between the onsets of the two diseases was 30 months (range 7 months to 20 years). The symptoms of MG included fatigable ptosis (64.7%), bulbar symptoms (58.8%), muscle fatigability (64.7%), diplopia (64.7%), dyspnea (23.5%), and facial paralysis (5.9%). Anti-acetylcholine receptor antibody was positive in 70.6% patients. All the patients had sicca symptoms. Manifestations of pSS also included swollen exocrine glands (23.5%), joint pain (23.5%), hair loss (11.8%), leukopenia (11.8%), recurrent oral ulcers (5.9%), Raynaud phenomenon (5.9%), and fever (5.9%). ANA positivity was present in 70.6% patients, anti-SSA positivity in 47.1%, and double positivity of anti-SSA and anti-SSB in 17.6%. There were 12 patients (70.6%) with two autoimmune diseases (pSS and MG), and five patients with more than two autoimmune diseases. Cholinesterase inhibitors were the most commonly prescribed drugs (82.4%). Seven patients received thymectomy and one patient improved after the operation. Two patients were given intravenous methylprednisolone pulse therapy, and four patients oral steroids combined with immunosuppressants initially. Intravenous immunoglobulin and plasma exchange were used in two patients, respectively, for the respiratory failure. All the patients improved following treatment except one patient who died of MG crisis due to medication withdrawal.

**Conclusion:** The coexistence of SS with MG is quite rare. The onset of MG may occur before or after the diagnosis of SS. Co-morbidity with MG does not seem to adversely affect the course of SS. Thus, controlling the progress of MG is the critical aspect of treatment.

## Introduction

Myasthenia gravis (MG) is a chronic autoimmune neuromuscular disease in which antibodies bind to acetylcholine receptors or to functionally related molecules in the post-synaptic membrane and cause weakness in the skeletal muscles resulting in difficulty in respiration and swallowing, diplopia, and ptosis (1). The weakness typically worsens with exercise and sustained muscle use and fluctuates over the course of a day. A few papers have noted that MG can be accompanied by concomitant autoimmune diseases (ADs), including thyroiditis, chronic inflammatory demyelinating polyradiculoneuropathy, neuromyelitis optica spectrum disease (NMOSD), and connective tissue diseases (CTD) (2). Among the CTDs, systemic lupus erythematosus (SLE) and rheumatoid arthritis (RA) are the most frequently mentioned (1). However, the association of MG with primary Sjögren's syndrome (SS) is unusual.

SS is a common multisystem autoimmune disease characterized by lymphocytic infiltration of exocrine glands. Patients often present with dry mouth and dry eyes due to hypofunction of salivary and lacrimal glands (3). It shows a female predominance of 9:1 and a peak incidence at the age of ~50 years (4). SS may occur in isolation or coexist with organ-specific autoimmune diseases (called as primary SS, pSS), such as thyroiditis and NMOSD (5). It also can be secondary to other systemic ADs, such as RA, SLE, or systemic sclerosis (SSc) (4). The nervous system is one of the targets of systemic damage in pSS patients (6). Neurologic manifestations of pSS are diverse, and may involve the peripheral nervous system and/or central nervous system (7). The coexistence of MG and pSS is limited to reports of one to two cases (8–21). Herein we report three cases of MG with pSS diagnosed at our hospital and review the relevant literature.

## Materials and Methods

### Case Presentation

#### Case 1

A 32-years-old Chinese woman presented with a 3-years history of bilateral fatigable ptosis and dysarthria and 1-year diplopia. She was admitted to the Department of Neurology of our hospital on June 12th, 2019, because of aggravation of her symptoms. She did not have difficulty in swallowing, shortness of breath, or muscle fatigability. The personal and family history was unremarkable. She had a positive response to the neostigmine test and the serum level of anti-acetylcholine receptor (AchR) antibody was >20 nmol/L (normal range 0–0.4 nmol/L). Computed tomography (CT) scan of the chest revealed a thymic remnant in the anterior mediastinum. She was diagnosed with MG according to definitions of MG (22). Meanwhile, the serum immunological examination was done routinely in order to screen for possible coexisting ADs. Surprisingly, the result of anti-nuclear antibody (ANA) spectrum showed that ANA was positive, with a titer of 1:3,200 and speckled pattern, and anti-SSA positive (++). Then the patient was transferred to our department to identify the rheumatic diseases. With regard to her illness history, the patient had suffered from dry mouth and dry eyes for half a year. Her dry mouth did not affect solid food intake. She denied having a rash, photosensitivity, oral ulcers, joint pain or Raynaud phenomenon. Blood routine tests showed a moderate leukopenia (white blood cells 2.59 × 10^9^/L). Urinary analysis was normal. Liver, renal functions and levels of creatine kinase (CK) were within normal range. Thyroid function normal. Erythrocyte sedimentation rate (ESR) and C reactive protein (CRP) normal. Immunoglobulin (Ig) G slightly high (17 g/L) and complements slightly low (C3 0.64 g/L, C4 0.14 g/L). Other autoantibodies including rheumatoid factor (RF), anti-cyclic citrullinated peptide (CCP) antibody, anti-cardiolipin antibody, anti-neutrophil cytoplasmic antibodies, the myositis-associated autoantibodies (MAA), and myositis-specific autoantibodies (MSA) including 16 different antigens (Mi-2α, Mi-2β, TIF1γ, MDA5, NXP2, SAE1, Ku, PMScl100, PM-Scl75, Jo-1, SRP, PL-7, PL-12, EJ, OJ, Ro-52) were all negative. The anti-thyroid peroxidase (TPO) antibody and anti-thyroglobulin (TG) antibody were also negative. Oral unstimulated salivary flow rate was 0.8 ml/min. Tests for dry eyes done by an ophthalmologist showed tear film break-up time (BUT) of left/right eye was 3.44/10.77 s, respectively; Schirmer test: left 2 mm/5 min, right 3 mm/5 min; Corneal fluorescence staining (–). Biopsy of the labial gland revealed focal lymphocytic sialadenitis with a focus score ≥1. Eventually, the patient was diagnosed with MG and pSS. She was given pyridostigmine bromide 60 mg three times per day, prednisone 30 mg per day, tacrolimus 1 mg twice per day. In a follow-up of 8 months, her symptoms improved obviously.

#### Case 2

A 55-years-old Chinese woman was admitted to our department due to dry mouth and dry eyes on October 11th, 2019. She suffered from dry mouth for 1 year but without decay of teeth or swelling of parotid glands. Two months before her admission, she began to have dry eyes and asymmetrical fatiguable ptosis. She also developed diplopia, dizziness and proximal muscle fatigability of lower limbs. She was identified as an asymptomatic hepatitis B virus carrier for 8 years. On admission, physical examination showed bilateral ptosis, more serious on the right side. Bilateral limitation of eye movement. No oral saliva pool. Muscle strength of proximal muscles of lower limbs was grade IV.

She was given a comprehensive laboratory examination involving blood cells, acute phase response markers, serum biochemistry, CK, immunoglobulins, autoantibodies, and hepatitis B virus markers. The clinically significant results showed as follows: hepatitis B surface antigen (HBsAg), hepatitis B e antibody (anti-HBe), and hepatitis B core antibody (anti-HBc) positive. The levels of CK was normal. RF was 76 IU/ml. The level of anti-TPO antibody was 78.6 IU/ml (0–9), anti-TG antibody 10.6 IU/ml (0–4), but thyroid function normal. The MAA and MSA were all negative. ANA was positive with a titer of 1:320 (homogeneous pattern). Ig G/A/M, complement C3/C4, ESR, and CRP were all normal.

**Figure 1:**
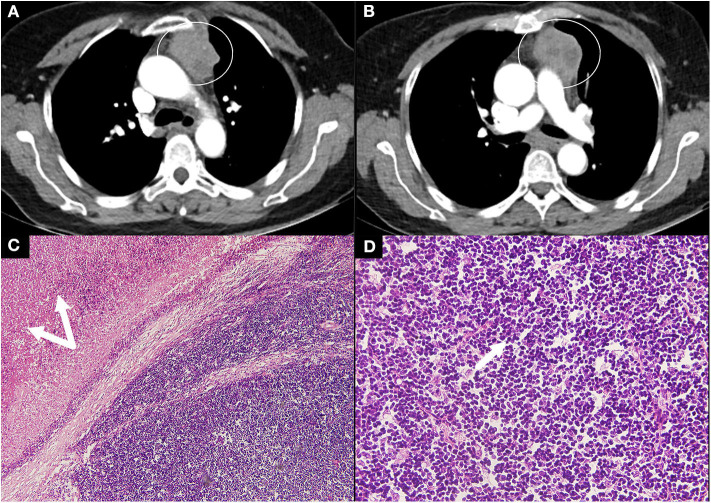
Contrast CT scan and thymoma pathology in case 2. **(A,B)** Contrast CT scan of the chest showed a large rounded mass (circle) with multiple low-density areas reflecting necrosis. **(C,D)** Pathology revealed type B1 thymoma according to the WHO classification with massive necrosis (double arrows, magnification, H&E ×10) and a predominance of lymphocytes (arrow, magnification, H&E ×40). CT, computed tomography; WHO, World Health Organization; H&E, hematoxylin and eosin.

She had a positive response to the neostigmine test and the serum level of anti-AchR antibody was 20 nmol/L. Thyroid ultrasound revealed a diffusely uneven echo pattern and a 0.8 × 0.4 cm hypoechoic nodule in the left lobe. Contrast-enhanced chest CT detected an anterior mediastinal mass indicating a thymoma (Figure 1). Oral unstimulated salivary flow rate was 0 ml/min. The presence of dry eyes was confirmed based on the ophthalmic examination. Biopsy of the labial gland revealed focal lymphocytic sialadenitis with a focus score ≥1. As a result, she was diagnosed with pSS, MG and Hashimoto's thyroiditis (HT). Thymectomy was performed and the post-operative pathology indicated Type B1 thymoma according to World Health Organization (WHO) classification (Figure 1). She was given pyridostigmine bromide 60 mg three times per day. In a follow-up of 6 months, her sicca and myasthenia symptoms were relieved.

#### Case 3

A 47-years-old Chinese woman was admitted to our department on November 7th, 2019. She suffered from dry mouth and dry eyes for 2 years. She also had hair loss, pain in knee and proximal interphalangeal joints without swelling. She developed fatigue and proximal muscle fatigability of lower limbs 1 year ago and diplopia 5 months ago. One month before admission, she began to have dysarthria, dysphagia, fatigability in chewing and facial paralysis. She had a 3-years history of Hashimoto's thyroiditis and hypothyroidism, and euthyrox was administered. Physical examination showed muscle strength of proximal muscles of lower limbs was grade IV. Laboratory tests showed as follows: white blood cells (WBC) 3.49 × 10^9^/L, IgG 25.6 g/L, ESR 30 mm/h, and CRP normal. RF was 21 IU/ml and anti-CCP antibody 104.07 U/ml. Thyroid function was normal, but the level of anti-TPO antibody was 10.4 IU/ml (0–9), anti-TG antibody negative. Serum level of anti-AchR antibody was 43.89 nmol/L. The levels of CK was normal. The MAA and MSA were all negative. ANA was 1:320 (+) (speckled pattern), anti-SSA (+++). Oral unstimulated salivary flow rate was 2.4 ml/min and dry eyes were confirmed by an ophthalmologist. Biopsy of the labial gland revealed focal lymphocytic sialadenitis with a focus score ≥1. Chest CT detected no thymus abnormalities.

The patient was diagnosed with pSS, MG, and HT. She was given pyridostigmine bromide 60 mg three times per day, prednisone 30 mg per day, and tacrolimus 3 mg per day. Her muscle fatigability, dysphonia and dysphagia soon alleviated. But 2 months later, she developed dyspnea when prednisone was tapered to 25 mg per day. Then intravenous immunoglobulin (IVIG) 20 g per day was administered for 5 days. Her dyspnea was relieved soon. Followed up for 3 months, her condition was stable.

## Methods

All analyses were based on a review of medical records that had been obtained for clinical purposes or for previous published studies, thus ethical approval was waived. To describe the clinical features, treatment regimens and outcome about patients with pSS and MG, we searched the PubMed, Medline databases and China Wanfang databases for reports of cases by using the keywords “Sjögren's syndrome,” “sicca syndrome,” “autoimmune diseases,” and “myasthenia gravis” in different combinations. Twenty-one cases with the two coexisting diseases were identified in total, and we reviewed the 14 cases written in English, Chinese, or Japanese with detailed data. The clinical features of MG patients with pSS were analyzed with descriptive statistics.

## Results

Through a comprehensive search of literature, we finally identified 14 other cases with MG and pSS (8–21). The details of the 17 patients (including three cases in the present study) are listed in Table 1.

**Table 1:** Review of present and previous reported cases with MG and pSS.

**Country**	**Sex/age**	**MG features**	**pSS features**	**Laboratory tests**	**Treatment**	**Outcome**
1966, United Kingdom (8)	F/51	Diplopia, dysphagia, muscle fatigability and dyspnea for 23 years; anti-AcR NA	Joint pain for 3 years, dry mouth for 3 months; Raynaud's phenomenon; keratoconjunctivitis sicca(+);	ESR elevated; ANA(+)	Cholinesterase inhibitor; thymectomy; analgesics	Improved
1973, Japan (9)	M/53	Dysphagia, muscle fatigability, and ptosis for 13 years; neostigmine test(+); anti-AcR NA; RNS(+); CT: thymic hypertrophy	Dry mouth for 1 year; Schirmer(+); salivary flow rate decreased; Sialography (+); Lip biopsy(+)	γ-globulin elevated; ESR normal, CRP elevated; RF(+); ANA(–); anti-SMA(+)	Cholinesterase inhibitor	Improved
1973, Scotland (10)	F/66	Ptosis, diplopia and generalized muscle weakness for 3 years; neostigmine test(+); anti-AcR NA	Left parotid gland enlargement, dry eyes for 2 years; Sialography (+); keratoconjunctivitis(+);	RF(+); ANA(+)	Cholinesterase inhibitor	Improved
1990, Sweden (11)	F/40	Muscle fatigability, dysarthria for 9 months; anti-AcR(+); tensilon test(+)	Transient dry mouth and a swelling below the mandible appeared after pyridostigmine bromide treatment; Schirmer normal; FL(+); salivary flow rate 1.8 ml/min; Lip biopsy(+)	WBC decreased; ESR elevated; RF(+); ANA(–)	Pyridostigmine bromide; thymectomy (thymic hyperplasia)	Improved; recurrent swollen exocrine glands
1999, Japan (12)	F/28	Exhaustion, ptosis and diplopia for 2.5 years; severe muscle fatigability and dyspnea for 1 year; anti-AcR (+); tensilon test(+); No thymic tumors	Sicca symptoms for 3 years; Swelling of bilateral parotid glands; Schirmer(+); salivary flow rate 0.5 ml/min; Sialography (+)	ANA, anti-SSA, anti-SSB(+)	Steroid pulse therapy; Pred 2 mg/kg daily; pyridostigmine bromide; 5 plasma exchanges	Improved
2003, China (13)	F/48	Muscle fatigability for 8 years; ptosis, mild diplopia and fatigue for 3 years; anti-AcR elevated; RNS(+)	Dry mouth, dry eyes, dental caries for 3 years; Schirmer (+); FL (+); salivary flow rate 0.5 ml/min	ESR 23 mm/h; CRP 1.61 mg/dl; anti-SSA, anti-SSB (–)	Cholinesterase inhibitor	Improved
2004, Japan (14)	F/36	Nausea, cough and high fever; chest CT: thymoma; 2 months post-thymectomy, bilateral ptosis, dysphagia and generalized fatigability developed; anti-AcR(+); tensilon test(+); RNS(+)	Dry mouth and dry eyes developed 1 month before surgery; Schirmer (+); salivary flow rate 0.15 g/2 min; lip biopsy (+); 11 months after surgery, PRCA occurred	γ-globulin elevated; ANA (+); anti-SSA, anti-SSB (-);	Thymectomy(type-B1 thymoma); local radiotherapy; prednisolone; PRCA: cyclosporin	Improved
2006, China (15)	F/28	Dysphagia for 1 year; fatigable ptosis for 3 weeks; neostigmine test(+); anti-AcR NA; fatigue test(+); RNS(+)	Dry mouth for 2 years; Schirmer (+)	RF (+); ANA: NA; anti-SSA (+)	Pred 1 mg/Kg/d	Improved
2006, China (16)	F/71	Muscle fatigability and mild diplopia for 4 years; neostigmine test(+); anti-AcR NA; fatigue test(+); RNS(+); pyridostigmine bromide 60 mg tid, improved; 3 days of dysphagia, worsening muscle fatigability, dyspnea, MG crisis	Dry mouth, dry eyes and joint pain for 5 years; FL (–); parotid ECT (+)	ESR elevated; γ-globulin elevated; ANA, anti-SSA, anti-SSB (–)	Cholinesterase inhibitor; tracheal intubation and ventilator assisted breathing	Die
2008, China (17)	F/41	Muscle fatigability and mild diplopia for 10 months; dysphagia for 3 months; anti-AcR(+); fatigue test(+); RNS(–)	Dry eyes and hair loss for 3 months; Schirmer (+); FL (+); parotid ECT (+)	IgG/A/M elevated; RF (+); ANA, anti-SSA (+)	Thymectomy(thymic hyperplasia); steroid + pyridostigmine bromide	Improved
2009, China (18)	F/78	Fatigable ptosis, diplopia and dysphagia for 7 years; anti-AcR(+); fatigue test(+); RNS(+); treatment: pred 60 mg qd → 15 mg qd and pyridostigmine bromide; improved	Dry mouth, dry eyes, joint pain; swelling of parotid glands and low grade fever for 1 year; Schirmer (+); salivary flow rate 0.05 ml/min	RF (+); ANA, anti-SSA, anti-SSB (+)	Pred 15 mg qd; pyridostigmine bromide	Improved
2013, China (19)	M/63	Productive cough, fatigability and ptosis for 3 months; anti-AcR(+); RNS(+); chest CT: thymoma	Dry eyes for 1 year; Schirmer (+); parotid ECT (+)	ANA (+)	Thymectomy(type-A thymoma)	Improved
2013, China (20)	F/46	Ptosis and mild diplopia for 2 months; neostigmine test(+); anti-AcR(+); fatigue test(+); RNS(+)	Dry mouth, dry eyes, oral ulcers and muscle pain of lower limbs for 2 years; Schirmer (+); FL (+); parotid ECT (+)	ESR 38 mm/h; RF (+); ANA, anti-SSA, anti-SSB (+)	Pred 50 mg qd; pyridostigmine bromide	Improved
2018, China (21)	F/52	Fatigable muscle fatigability for 11 years, 10 years ago, dyspnea developed; anti-AcR(+); fatigue test(+); pred and pyridostigmine bromide relieve her symptoms. improvement of muscle fatigability post-thymectomy	Dry mouth and dry eyes for 9 years; parotid ECT (+)	RF (+); ANA, anti-SSA (+); Anti-TPO, anti-TG (+); Anti-AQP4-IgG (+)	MP pulse → pred; MMF	Improved
Case 1	F/32	Ptosis and dysarthria for 3 years, diplopia for 1 year; neostigmine test(+); anti-AcR(+); RNS(+)	6 months of dry mouth and dry eyes; Schirmer:(+); FL(-); salivary flow rate 0.8 ml/min; lip biopsy (+)	WBC decreased; IgG elevated; ANA, anti-SSA(+)	Pyridostigmine bromide; pred; tacrolimus	Improved
Case 2	F/55	Ptosis, diplopia and muscle fatigability for 1 month; neostigmine test(+); anti-AcR(+); RNS(+); chest CT: anterior mediastinal mass	Dry mouth for 1 year; dry eyes for 2 months; Schirmer:(+); FL(+); salivary flow rate 0 ml/5 min; lip biopsy(+)	RF(+); ANA(+); Anti-SSA, anti-SSB(–)	Thymectomy (type-B1 thymoma); pyridostigmine bromide	Improved
Case 3	F/47	Muscle fatigability for 1 year, diplopia for 5 months; dysarthria, dysphagia, fatiguable chewing and facial paralysis for 1 month; anti-AcR(+); fatigue test(+); RNS(+)	Dry mouth, dry eyes, hair loss and joint pain for 2 years; Schirmer(+); salivary flow rate 2.4 ml/min; lip biopsy(+)	WBC decreased; IgG elevated; ESR 30 mm/h; RF, anti-CCP(+); ANA, anti-SSA(+)	Pyridostigmine bromide; pred; tacrolimus; MG crisis: IVIG	Improved

*MG, myasthenia gravis; pSS, primary Sjögren's syndrome; F, female; M, male; anti-AcR, anti-acetylcholine receptor; FL, corneal fluorescence stain; RF, rheumatoid factor; ANA, anti-nuclear antibody; pred, prednisone; RNS, repetitive nerve stimulation test; ESR, erythrocyte sedimentation rate; CRP, C reactive protein; PRCA, pure red cell aplasia; NA, not available; ECT, emission computed tomography; Ig, immunoglobulin; anti-TPO, anti-thyroid peroxidase; anti-TG, anti-thyroglobulin; MP, methylprednisolone; MMF, mycophenolate mofetil; anti-AQP4, anti- aquaporin 4; WBC, white blood cells; anti-CCP, anti-cyclic citrullinated peptide; IVIG, intravenous immunoglobulin*.

The demographic features, precedence of disease development, common manifestations, autoimmune complications, treatment regimens, and outcome of all 17 patients are reviewed in Table 2. Notably, anti-AcR antibody were positive in 12 out of 17 cases (unclear in the other five patients). Most patients (16 out of 17 cases) were classified as generalized MG presenting with limb muscle fatigability or bulbar symptoms while only one patient was ocular MG. Of seven patients who underwent thymectomy, five had pathologic results: three had thymoma (two with type B1, one with type A according to the WHO classification), and two thymic hyperplasia. Additionally, there were six patients who had lip biopsy and all the results revealed focal lymphocytic infiltration. Interestingly, there were five patients having more than two ADs besides MG and pSS. With regard to treatment, thymectomy relieved the symptoms obviously in the patient with thymoma. Cholinesterase inhibitors were the most commonly used drugs, and three patients were treated efficiently with the drug alone. Other therapeutic methods, including intravenous methylprednisolone pulse, oral steroids, immunosuppressants, plasma exchange (PLEX), and IVIG were also used. Overall, all the patients improved following the treatment except one patient who died of MG crisis due to medication withdrawal by herself.

**Table 2:** Features of patients with MG and pSS.

**Items**	**Features**
Median age at onset (years, y)	48 (28~78) y
Gender ratio, female:male	15:2
Initial onset of MG	9/17 (52.9%)
**Median duration**	
MG to pSS (months, m)	30 m (7 m to 20 y)
pSS to MG (months, m)	11.5 m (3 m to 22 m)
**MG manifestations**	
Fatigable ptosis	11/17 (64.7%)
Bulbar symptoms	10/17 (58.8%)
Muscle fatigability	11/17 (64.7%)
Diplopia	11/17 (64.7%)
Dyspnea	4/17 (23.5%)
Facial paralysis	1/17 (5.9%)
Anti-AcR antibody (+)	12/17 (70.6%)
**pSS manifestations**	
Sicca symptoms	17/17 (100%)
Swollen exocrine glands	4/17 (23.5%)
Joint pain	4/17 (23.5%)
Hair loss	2/17 (11.8%)
Recurrent oral ulcers	1/17 (5.9%)
Raynaud phenomenon	1/17 (5.9%)
Fever	1/17 (5.9%)
Decreased WBC	2/17 (11.8%)
ANA (+)	12/17 (70.6%)
Anti-SSA (+)	8/17 (47.1%)
Anti-SSA and anti-SSB (+)	3/17 (17.6%)
Rheumatoid factor (+)	9/17 (52.9%)
Anti-CCP antibody (+)	1/17 (5.9%)
Lip biopsy (+)	6/6 (100%)
**Polyautoimmuty**	
pSS, MG, and HT	3/17 (17.6%)
pSS, MG, and thymoma	3/17 (17.6%)
pSS, MG, and NMOSD	1/17 (5.9%)
pSS, MG, and PRCA	1/17 (5.9%)
**Treatment**	
Cholinesterase inhibitor	14/17 (82.4%)
Steroids	9/17 (52.9%)
Immunosuppressants	4/17 (23.5%)
IVIG	1/17 (5.9%)
Plasma exchange	1/17 (5.9%)
Thymectomy	7/17 (41.2%)
**Outcome**	
Improved	16/17 (94.1%)
Died	1/17 (5.9%)

*MG, myasthenia gravis; pSS, primary Sjögren's syndrome; anti-AcR, anti-acetylcholine receptor; WBC, white blood cells; ANA, anti-nuclear antibody; anti-CCP, anti-cyclic citrullinated peptide; HT, Hashimoto's thyroiditis; NMOSD, neuromyelitis optica spectrum disorder; PRCA, pure red cell aplasia; IVIG, intravenous immunoglobulin*.

## Discussion

MG is a B-cell mediated organ-specific autoimmune disease with antibodies against the acetylcholine receptor, muscle-specific kinase (MUSK), lipoprotein-related protein 4 (LRP4), or agrin in the post-synaptic membrane at the neuromuscular junction (23). About 10% MG patients may have a thymoma, and conversely, one third of patients with thymoma can develop MG (1). Similarly, pSS is also a B-cell mediated systemic autoimmune disease with multiple antibodies including ANA, anti-SSA (Ro) antibody and anti-SSB (La) antibody. Thereafter, there might be a similar immunologic mechanism involving different targets shared by these two diseases. The overall prevalence of neurologic involvement in pSS is ~20% (24). The neurologic manifestations include peripheral neuropathy, aseptic meningitis, NMOSD, and multiple sclerosis-like manifestations. But the coexistence of pSS and MG is really rare and limited to case reports.

Herein we present three cases of MG coexisting with pSS and offer a review based on the published literature. We notice that MG has rarely been reported to coexist with pSS. But its incidence may have been underestimated, because the sicca symptoms are easily overlooked by a neurologist. The coexistence of MG and pSS show a female predominance and a median age of 48 years at onset, which are consistent with the features of pSS and the age of early onset MG (EOMG). MG occurred before pSS in more than half of the patients. Thus, it is of great importance for a neurologist to screen patients with MG for the presence of other autoimmune rheumatic disorders including pSS.

The frequency of coexistence with other ADs in MG patients has been reported between 11.6 and 32% (2). Data from a large population-based survey showed that 214 ADs were diagnosed in 185 of 984 MG patients (18.8%). And 26 of these subjects had two or more ADs (25). Autoimmune thyroid diseases (AITD) is the most common coexisting condition, followed by SLE and RA. Other autoimmune disorders including chronic inflammatory demyelinating polyradiculoneuropathy and NMOSD were also reported. Furthermore, inflammatory myopathy is another rare AD which can coexist with MG. Garibaldi et al. observed that 13 out of 441 (2.9%) MG patients developed myositis and 10/13 patients occurred simultaneously (26). So the authors recommended myositis should be considered when MG patients had the features of elevated serum CK levels or stable muscle weakness unresponsive to acetylcholinesterase inhibitors (26), in particular for MG patients with thymoma (27). As for the three patients we reported, they all had normal serum CK levels and responded well to the acetylcholinesterase inhibitors. As a result, myositis was ruled out.

Actually, the term “polyautoimmunity” has been used for decades and defined as the presence of two or more ADs in an individual. In this paper, there are 12 patients (70.6%) having two ADs (pSS and MG), and five patients having more than two ADs. Moreover, one patient suffered from four ADs simultaneously. On the other hand, pSS often occurs with organ-specific ADs and the most common coexisting AD is also AITD (28). The prevalence of AITD was 11.1–15.7% in patients with pSS (29, 30). Moreover, a study by Lazarus reported that 7.9% of patients with pSS had two or more ADs (31).

Since the coexistence of MG and pSS is quite rare, the pathogenesis remains elusive. Berrih-Aknin summarized the common mechanisms between MG and SS (32). The frequency of human leukocyte antigen (HLA)-DR3 in the whole group of MG patients was increased compared with the control population, and the same HLA haplotype was one of the loci for susceptibility for SS. The EOMG patients were predominantly women (female to male 9:1) with thymic hyperplasia, including the development of ectopic germinal centers in the thymus and high levels of anti-AChR antibody. Sex hormones which may affect both innate and adaptive immune systems are mainly thought to be responsible for this bias. And the formation of ectopic germinal centers in salivary glands was also commonly found in SS patients (32). Serum levels of both B cell activating factor (BAFF) and a proliferation-inducing ligand (APRIL) increased in SS and MG patients (32). In addition, increased expression of interleukin-17 was found in the sera and the thymus of anti-AcR positive MG patients as well as in the saliva of SS (32). These findings highlight the key role of the target organ in the initiation and development of the disease.

It is noticed that although all 17 pSS patients with MG presented with sicca symptoms, no one developed severe systemic damage as far as pSS is concerned (four patients with swollen exocrine glands, four with joint pain, two with slightly decreased WBC). Therefore, no steroids or immunosuppressants were given for treating pSS. Topically symptomatic treatments were used as the first-line therapy for oral and ocular dryness based on the European League Against Rheumatism (EULAR) recommendations for the management of pSS (33). The treatment regimens in these coexisting conditions were mainly aimed at MG. Cholinesterase inhibitors were the most common drugs for alleviating the symptoms of MG with affirmative effect (34). For patients with MG who did not respond to an adequate trial of pyridostigmine, corticosteroids could be recommended. Attention should be paid to the precaution that the dose of corticosteroids should be increased gradually to avoid an initial deterioration (35). Immunosuppressive agents were recommended to be used alone or used as corticosteroid-sparing agents, when corticosteroids were contraindicated or refused (35). Among the 17 patients, there were two patients given intravenous methylprednisolone pulse therapy with subsequent oral steroids. And four patients took oral steroids combined with immunosuppressants initially. They all had a good response. IVIG and PLEX could be used in pSS patients with severe systemic involvements, such as severe thrombocytopenia or in MG patients with life-threatening signs, such as respiratory insufficiency or dysphagia (33, 34). The effect of IVIG and PLEX treatment was seen in two patients who developed MG crisis with respiratory difficulty (one treated with IVIG, and the other with PLEX). In addition, thymectomy is a special option, not only for the patients with a thymoma, but also for the non-thymomatous anti-AChR antibody positive MG (36). Thymectomy should be considered early in treatment decisions to improve clinical status as the thymus is thought to be a major trigger of autoantibody production (36). We observed that there were seven patients with MG and pSS who received thymectomy, and one patient improved after thymoma resection without any medication.

A single-center retrospective study showed rituximab was effective in patients with MG, supporting the role of B cell depletion in the management of MG (37). It has been reported that rituximab may be a useful treatment for pSS (33). Thus, it is reasonable to speculate that rituximab may be effective for the co-morbidity of MG and pSS. However, no such patient has been treated with rituximab, to date.

Another question is whether hydroxychloroquine (HCQ) can be used in patients with pSS and MG. HCQ is an essential drug for patients with CTDs including SLE and SS, but special caution should be taken when used in MG patients. Although data from the Spanish society of Rheumatology Lupus Registry showed that HCQ protected against polyautoimmunity for patients with SLE (38), Varan et al. reported a case of SLE in which MG developed with the use of HCQ, and regressed with its withdrawal (39). In a series of 17 patients with SLE and MG, Jallouli et al. found that eight patients (47%) developed MG after initiation of HCQ, but only one patient who received HCQ had an exacerbation of myasthenic symptoms (40). It is worth mentioning that MG has long been recognized as one of the 19 neuropsychiatric manifestations of SLE (41). Therefore, whether it is caused by HCQ or associated with SLE is worth further research. As for the effect of HCQ on the patients with both pSS and MG, there has been no such report to date.

## Conclusion

The coexistence of MG with pSS is quite rare according to the reported cases. The onset of MG may occur before or after the diagnosis of pSS. It is of great importance to screen for ANA during the clinical course of MG, and to screen for MG when pSS patients complain of muscle fatigability or fatigable ptosis. Severe morbidity due to pSS is uncommon in patients with both diseases. Thus, controlling the progress of MG is the critical aspect of treatment. Therapeutic decisions should be made following a multidisciplinary approach. Multicenter prospective studies of larger sample sizes are needed to achieve a better understanding of this co-morbidity.

## Data Availability Statement

The raw data supporting the conclusions of this article will be made available by the authors, without undue reservation.

## Ethics Statement

The written informed consent was obtained from the participants for the publication of this paper.

## Author Contributions

XL and YZ collected the patients' data and designed the study. XL wrote the manuscript. YZ, QL, and YD critically revised the manuscript. All authors read and approved the submitted version.

## Conflict of Interest

The authors declare that the research was conducted in the absence of any commercial or financial relationships that could be construed as a potential conflict of interest.
